# Developing a method to derive alcohol-attributable fractions for HIV/AIDS mortality based on alcohol's impact on adherence to antiretroviral medication

**DOI:** 10.1186/1478-7954-9-5

**Published:** 2011-02-14

**Authors:** Gerrit Gmel, Kevin D Shield, Jürgen Rehm

**Affiliations:** 1 Centre for Addiction and Mental Health (CAMH), 33 Russell Street, Toronto, Ontario, M5S 2S1, Canada; 2 Ecole Polytechnique Fédérale de Lausanne, Route Cantonale, 1015 Lausanne, Switzerland; 3 Dalla Lana School of Public Health (DLSPH), University of Toronto, 6thFloor, Health Sciences Building 155 College Street, Toronto, Ontario, M5T 3M7, Canada; 4 Institute for Clinical Psychology and Psychotherapy, Technische Universität Dresden, Chemnitzer Str. 46, D-01187 Dresden, Germany

## Abstract

**Background:**

Alcohol consumption is causally linked to nonadherence to antiretroviral treatment that in turn causes an increase in HIV/AIDS mortality. This article presents a method to calculate the percentage of HIV/AIDS deaths attributable to alcohol consumption and the associated uncertainty.

**Methods:**

By combining information on risk relations from a number of published sources, we estimated alcohol-attributable fractions (AAFs) of HIV/AIDS in a stepwise procedure. First, we estimated the effect of alcohol consumption on adherence to antiretroviral treatment, and then we combined this estimate with the impact of nonadherence on death. The 95% uncertainty intervals were computed by estimating the variance of the AAFs using Taylor series expansions of one and multiple variables. AAFs were determined for each of the five Global Burden of Disease regions of Africa, based on country-specific treatment and alcohol consumption data from 2005.

**Results:**

The effects of alcohol on HIV/AIDS in the African Global Burden of Disease regions range from 0.03% to 0.34% for men and from 0% to 0.17% for women, depending on region and age category. The detrimental effect of alcohol consumption was statistically significant in every region and age category except for the North Africa/Middle East region.

**Conclusions:**

Although the method has its limitations, it was shown to be feasible and provided estimates of the impact of alcohol use on the mortality outcome of HIV/AIDS.

## Background

Alcohol has been identified as a major risk factor for mortality and burden of disease in past comparative risk assessments within Global Burden of Disease studies [1,2]. In past iterations of comparative risk assessments, infectious diseases have not been included. However, evidence has been accumulating that alcohol has a causal impact on infectious disease categories [3,4].

Evidence indicates a strong association between alcohol and HIV/AIDS [5,6], but personality variables such as risk-taking or impulsive behavior cannot be excluded as potential alternative explanations [6]. There is sufficient evidence, however, that alcohol worsens the course of the disease, especially by impacting adherence to antiretroviral treatment. Globally, HIV/AIDS led to about 2 million deaths and 58.5 million lost disability-adjusted life years (DALYs) in 2004 [7]. Failing to estimate the alcohol-attributable HIV/AIDS burden could lead to substantial underestimation of the burden of disease and mortality attributable to alcohol.

Antiretroviral therapy has led to a change in the natural history of HIV [8-10]. Lima and colleagues have shown that nonadherence to antiretroviral therapy by as little as 5% has a significant effect on the mortality of HIV-infected people in a high-income country [11]. Studies from low- to middle-income countries have shown similarly elevated risks [12]. Therefore, adherence is a key to the success of antiretroviral therapy, and adherence levels of at least 90% to 95% are generally necessary to maximize treatment benefits [13,14]. Poor adherence to antiretroviral therapy is associated with an increased likelihood of hospitalization [15], as well as increased mortality [3,11,12,16].

Adherence is impacted by multiple variables such as injection drug use, forgetfulness, suspicions about treatment, complicated dosing regimens, number of pills required, decreased quality of life, and work and family responsibilities [17]. Alcohol use, and especially occasions of heavy drinking, has been shown to have a marked impact on adherence by interfering with one's capacity to plan for or remember dosing requirements. In addition, alcohol users might have decreased access to antiretroviral therapy or may use alcohol to reduce or avoid HIV-related negative mood states [18]. A recent meta-analysis [18] indicated that, compared to abstainers, drinkers have an odds ratio of 0.604 (95% confidence interval [CI]: 0.531, 0.687) of adhering at least 95% of the time to their treatment, meaning that those who used alcohol were about 0.60 times as likely to be classified as adherent to treatment as nonusers. Overall, the effect of alcohol use on antiretroviral treatment has been found to be causal [3,19].

**Figure 1:**
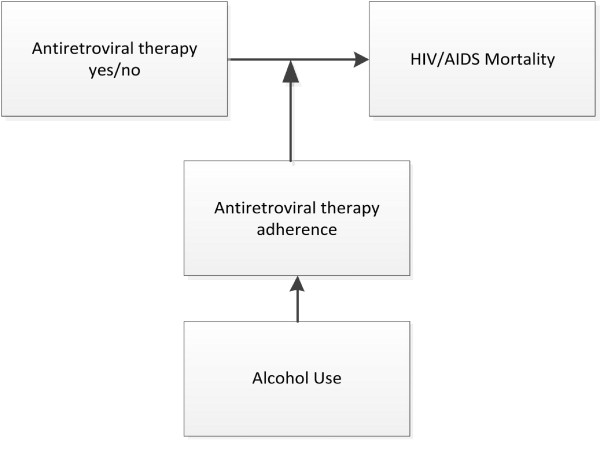
**Causal assumptions of our method of calculating mortality due to nonadherence to antiretroviral therapy because of alcohol use**.

Despite the demonstrated role of alcohol use on antiretroviral medication adherence, research on and modeling of the effect of alcohol use on the HIV/AIDS burden of mortality and disease remain limited [20]. This article suggests a method to quantify the fraction of HIV/AIDS deaths attributable to alcohol consumption from nonadherence to antiretroviral therapy. We estimate the alcohol-attributable fraction (AAF) for HIV/AIDS-related mortality by combining the effect of alcohol consumption on antiretroviral adherence and the effect of adherence on mortality. Therefore, this method only considers increased mortality due to alcohol consumption that is linked to a resulting worsened adherence to antiretroviral treatment. It does not take into consideration any effect that alcohol might have on the outcome of HIV/AIDS in the absence of treatment. These causal assumptions are summarized in Figure 1.

## Methods

We conducted an analysis using data from the five Global Burden of Disease (GBD) regions of Africa. These regions were chosen for their high variation in both prevalence of HIV/AIDS and adult per capita estimates of alcohol consumption. These regions are defined as follows:

• North Africa/Middle East: Algeria, Bahrain, Egypt, Iran, Iraq, Jordan, Kuwait, Lebanon, Libya, Morocco, Occupied Palestinian Territory, Oman, Qatar, Saudi Arabia, Syria, Tunisia, Turkey, United Arab Emirates, Western Sahara, Yemen

• Sub-Saharan Africa, Central: Angola, Central African Republic, Congo, the Democratic Republic of the Congo, Equatorial Guinea, Gabon

• Sub-Saharan Africa, East: Burundi, Comoros, Djibouti, Eritrea, Ethiopia, Kenya, Madagascar, Malawi, Mayotte, Mozambique, Rwanda, Somalia, Sudan, Tanzania, Uganda, Zambia

• Sub-Saharan Africa, South: Botswana, Lesotho, Namibia, South Africa, Swaziland, Zimbabwe

• Sub-Saharan Africa, West: Benin, Burkina Faso, Cameroon, Cape Verde, Chad, Côte d'Ivoire, Gambia, Ghana, Guinea, Guinea-Bissau, Liberia, Mali, Mauritania, Niger, Nigeria, Saint Helena, Sao Tome and Principe, Senegal, Sierra Leone, Togo

### Data sources

Country data on the proportion of people who are in need of antiretroviral therapy and receive such treatment were obtained for 2005 from the *2006 Report on the Global AIDS Epidemic *[21]. Regional data were then calculated as a population-weighted average. Table 1 outlines the resulting regional proportion of people receiving antiretroviral treatment compared to all those who are in need of such treatment for the five African GBD regions.

**Table 1:** Proportion of people in need of antiretroviral therapy receiving antiretroviral therapy.

Region	Proportion		
	
	lower bound	point estimate	upper bound
North Africa/Middle East	15.01%	17.36%	19.71%

Sub-Saharan Africa, Central	3.00%	4.25%	5.50%

Sub-Saharan Africa, East	12.07%	14.24%	16.41%

Sub-Saharan Africa, South	19.11%	21.67%	24.22%

Sub-Saharan Africa, West	11.36%	13.47%	15.59%

In order to establish the prevalence of nonadherence, we adopted an estimate of 40.1% for the rate of nonadherence to antiretroviral treatment (95% CI: 36.9%, 43.3%), provided by Lima and colleagues [18,22]. This estimate was between two other estimates of 47.6% and 31.8% for adherence rates of <80% and <90%, respectively [12,16]. Please note that even though the relative risk for people adhering to antiretroviral treatment compared to people who are nonadherent is larger than the relative risk for not being in treatment compared to being in treatment, this does not mean that being in antiretroviral treatment and partially adhering is associated with worse outcomes than not being in treatment at all. On the contrary, the risk relations between the different groups based on the assumptions of Table 2 are as follows:

**Table 2:** Parameters used in calculating the AAF for people who die of AIDS due to nonadherence to antiretroviral therapy.

Parameter	Symbol	Value	95% Confidence Interval	Source
Proportion of people on antiretroviral therapy who are not adhering to medication regimen	P_na_	40.1%	(36.9% to 43.3%)	Lima and colleagues [22]

Risk ratio of nonadherence to antiretroviral therapy, comparing current drinkers to abstainers	RR_drink_	1.82	(1.63 to 2.04)	Hendershot and colleagues[18]

Mortality risk ratio for nonadherence to antiretroviral therapy compared to those who adhere to antiretroviral therapy	RR_na_	3.13	(1.95 to 5.05)	Lima and colleagues[11]

Mortality hazard ratio for people not on antiretroviral therapy treatment compared to those who are	HR_nontreat_	2.63	(1.92 to 3.57)	Murphy and colleagues[23]

1 = risk of people in antiretroviral treatment adhering on an arbitrary scale

3.13 = risk of people in antiretroviral treatment not adhering to treatment

1.85 = risk of people in antiretroviral treatment overall (assuming 0.401 nonadherent)

4.88 = risk of people not in treatment

Multiple sources were used to characterize the relationship between alcohol consumption and nonadherence to antiretroviral therapy and the effect of nonadherence on HIV/AIDS-related mortality. To characterize the effect of alcohol consumption on antiretroviral adherence, we used the odds ratio of 0.60 (95% CI: 0.53, 0.69) from the meta-analysis performed by Hendershot and colleagues [18,22] for adherence by global drinkers (defined as alcohol consumption within the past month) compared to abstainers. Since there have been no meta-analyses of nonadherence on mortality, we used a hazard ratio of 3.13 (95% CI: 1.95, 5.05) for deaths of people not adhering to treatment (defined as less than 95% of the time) compared to people adhering to treatment, as reported by Lima and colleagues [11]. This estimate is similar to but slightly lower than the hazard ratios of 3.2 and 3.9 for adherence rates of <80% and <90%, respectively, as described in other studies [12,16]. For the rate ratio of HIV/AIDS-related deaths of people on antiretroviral treatment compared to people not on treatment, we used a rate ratio reported by Murphy and colleagues [23] that gives a point estimate of 0.38 (95% CI: 0.28, 0.52). These values were combined to obtain the AAF for HIV/AIDS for a given population. The following sections will first present the derivation of the AAF for HIV/AIDS mortality from nonadherence to antiretroviral therapy and then describe the derivation of the 95% uncertainty intervals associated with these AAFs. Estimations of uncertainty for the calculated AAFs are of critical importance for the estimated burden of disease attributable to alcohol and for policy recommendations aimed at reducing this burden. Table 2 outlines the measures of association used to compute the AAFs for the five African GBD regions and the prevalence of nonadherence to antiretroviral therapy for those who are currently receiving antiretroviral therapy.

### Derivation of AAFs for HIV/AIDS

Continuous modeling of the effects of alcohol use on adherence [24,25] was impossible given the data available to us, and thus we limited our analysis to two broad categories: drinkers versus nondrinkers, and antiretroviral therapy adherence (at least 95% of the time) versus nonadherence.

The attributable fraction (AF) is defined as:

$$AF\;=\;\frac{P\left(RR\;-\;1\right)}{P\left(RR\;-\;1\right)\;+\;1}$$

where P represents prevalence of exposure and RR represents the relative risk for exposure compared to no exposure. Depending on the design of the original epidemiological study, the RR information could be derived from hazard ratios, risk ratios, or odds ratios.

However, for the AAF of mortality due to not adhering to antiretroviral therapy for people with HIV/AIDS, the lack of research and limited information available on the effects of alcohol on HIV/AIDS forced us to calculate the AAFs for HIV/AIDS-related mortality in several steps (for the definition and calculation of AAF in general, see [26,27]).

First, we calculated the adherence attributable fraction (AdAF) using a definition of nonadherence as adherence to antiretroviral therapy less than 95% of the time. The AdAF represents the fraction of HIV/AIDS-related deaths attributable to nonadherence to antiretroviral therapy, and is calculated as follows:

$$AdAF\;=\;\frac{P_{adher}\;+\;P_{na}RR_{na}\;-\;1}{P_{adher}\;+\;P_{na}RR_{na}}$$

where P_adher _is the proportion of people adhering to treatment more than 95% of the time, P_na _is the proportion of people adhering to treatment less than 95% of the time, and RR_na _is the relative risk of mortality for those who are not adhering compared to those who are.

Second, we calculated the nonadherence due to alcohol attributable fraction (NAAAF) - the proportion of nonadherence due to alcohol consumption - as follows:

$$NAAAF\;=\;\frac{P_{abs}\;+\;P_{drink}RR_{drink}\;-\;1}{P_{abs}\;+\;P_{drink}RR_{drink}}$$

where P_drink _is the proportion of drinkers among the antiretroviral-treated HIV-infected population, P_abs _is the proportion of abstainers, and RR_drink _is the relative risk of nonadherence for drinkers compared to nondrinkers. Table 3 outlines the prevalence of current drinkers by age and sex for the five African GBD regions.

**Table 3:** Prevalence of current drinkers by region.

	Current Drinkers					
	
Region	Men (age in years)			Women (age in years)		
	
	15 - 34	35 - 54	55+	15 - 34	35 - 54	55+
North Africa/Middle East	7.2%	12.0%	5.5%	3.5%	1.1%	0.4%

Sub-Saharan Africa, Central	51.0%	51.9%	20.5%	32.4%	28.5%	11.9%

Sub-Saharan Africa, East	24.6%	38.8%	37.2%	16.1%	24.9%	21.4%

Sub-Saharan Africa, South	38.0%	38.0%	29.5%	12.6%	15.7%	9.8%

Sub-Saharan Africa, West	36.2%	50.9%	40.5%	20.8%	31.2%	27.1%

Finally, we needed to know the proportion of deaths of people under treatment compared to the total number of deaths in the population due to HIV infection. Because the exact numbers are not known, we estimated this proportion using the hazard ratio for deaths in people without treatment compared to people with treatment. This proportion of deaths of people undergoing treatment (PDT) was calculated as follows:

$$PDT\;=\;\frac{P_{treat}}{P_{treat}\;+\;HR_{nontreat}P_{nontreat}}$$

where P_treat _is the proportion of HIV-infected people receiving treatment, P_nontreat _is the proportion of HIV-infected people not receiving treatment, and HR_nontreat _is the hazard ratio of people without treatment. It should be noted that hazard ratio and relative risk are synonymous in this case. These three proportions can be combined to calculate the AAF for HIV/AIDS mortality for a given population.

AAF = AdAF ⋅ NAAAF ⋅ PDT

### Estimation of the variance of AAFs

It is possible to derive a mathematical expression of the variance of the AAFs when the variances of each parameter making up the final expression are known. Calculating the variance of the AAF of HIV/AIDS-related mortality requires the combination of several steps. Because these steps involve Taylor series expansions, they yield only an approximation of the real variance. We calculated the variance of the AAFs by calculating the variance of the simplest combinations first, which were then used in more complex functions. The derivations of the variance of two distributions and the derivation of the variances using Taylor series expansions can be found in 'additional file 1 appendix 1'.

In the literature, the uncertainty intervals for AFs due to risk factors based on meta-analyses are usually computed using a Monte Carlo-type method (for alcohol, see [3]). The Monte Carlo method uses repeated random samples of an AF to estimate its variance [28].

The AAF samples are obtained by randomly generating each parameter using the information we have on its distribution. The main advantage of this method is its broad range of applications. The method can be applied to virtually any field and any function and is especially useful in cases in which an algebraic solution to a problem is impossible to derive. Our AAF, however, is a relatively simple function of several independent variables that makes the use of Monte Carlo samples unnecessary. Indeed, an algebraic solution, when possible to obtain, should always be preferred to a simulation, as the results are more precise and require fewer resources. In fact, the results obtained with this method are generated instantaneously on any computer and do not require the use of servers or the handling of large datasets, as is usually the case with a Monte Carlo analysis. In addition, a decomposition of sources of variation and their relative contribution to overall variance can be obtained more readily. Even though it is possible to derive only an approximation of the variance of the original function using second order Taylor series expansions, the assumption that the spread of the function around its mean value is relatively small guarantees an accurate result. The variations around the point estimate of each AF function depend only on the variance of the parameters used; therefore, knowledge of these parameters is important. It should be noted that for large variances of each parameter, the error in the variance of the final function will increase as the assumption of small variations is not satisfied. This is not specific to the analytic expression derived here, but would also affect a Monte Carlo method for which a convergence would not be ensured unless a very large number of data points are collected [29]. Finally, the use of a second order approximation has the advantage of yielding very simple expressions that combine only the derivative of the function and the variance of its parameters; this means that they can be computed nearly instantaneously in R using only a few command lines. It should be noted that in many fields, such as electrical engineering, quantum mechanics, and protein engineering, Taylor series of order two or fewer are used daily and yield excellent results.

### Variance of AdAF and NAAAF

Using the variances of P_na _and RR_na _described in the previous section, we derived a mathematical expression for the variance of AdAF and NAAAF using Taylor series expansions. The complete derivations can be found in 'additional file 1, appendix1', and the final expression of the variance of these terms is as follows:

For AdAF:

$$\begin{array}{l} Var[AdAF(P_{na},RR_{na})]\;≅\\ \frac{1}{{\left(1\;+\;P_{na}(RR_{na}\;-\;1)\right)}^{4}}Var[P_{na}RR_{na}] \end{array}$$

where

$$\begin{array}{l} Var[P_{na},RR_{na}]\;\;=\;Var[P_{na}]Var[RR_{na}]\;+\\ RR_{na}{}^{2}Var[P_{na}]\;+\;P_{na}{}^{2}Var[RR_{na}] \end{array}$$

And for NAAAF:

$$\begin{array}{l} Var[NAAAF(P_{drink},RR_{drink})]\;≅\\ \frac{1}{{\left(1\;+\;P_{drink}(RR_{drink}\;-\;1)\right)}^{4}}Var[P_{drink}RR_{drink}] \end{array}$$

where

$$\begin{array}{l} Var[P_{drink}RR_{drink}]\;=\\ Var[P_{drink}]Var[RR_{drink}]\;+\\ RR_{drink}{}^{2}Var[P_{drink}]\;+\;P_{drink}{}^{2}Var[RR_{drink}] \end{array}$$

### Variance of PDT

The variance of the proportion of deaths of HIV-infected people under treatment (PDT) cannot be derived using the above methods. Since the variance of PDT is a function of two variables, we used a Taylor series expansion performed in two dimensions. These derivations are shown in additional file 1, appedx1, and the final expression of the variance is as follows:

$$\begin{array}{l} Var\left[PDT[P_{treat},HR_{non-treat}]\right]\;≅\\ D_{P}{{}_{{}_{treat}}}^{2}Var[P_{treat}]\;\;+\\ D_{HR_{nontreat}}{}^{2}Var[HR_{nontreat}] \end{array}$$

where D_Ptreat _and D_HRnontreat _are the partial derivatives of the PDT function with respect to P_treat _and HR_nontreat _and can be expressed as follows:

$$\begin{array}{l} D_{P_{treat}}\;=\\ \frac{HR_{nontreat}}{{\left(P_{treat}\;+\;HR_{nontreat}(1\;-\;P_{treat})\right)}^{2}}\\ D_{HR_{non-treat}}\;=\\ \;\frac{P_{treat}(1\;-\;P_{treat})}{{\left(P_{treat}\;+\;HR_{nontreat}(1\;-\;P_{treat})\right)}^{2}} \end{array}$$

In this manner, the variances of all three components of the final AAF function are determined. Using the variances for AdAF, NAAAF, and PDT, the variance of the AAF can be determined by the variance of (AdAF·NAAAF) PDT (see 'additional file 1, appendix 1').

### Computation of variances for hazard ratios and relative risks

Little information on the relative risks and hazard ratios mentioned above is available in the literature. Accordingly, previously published raw data had to be transformed to be useful for our analysis. The following paragraphs describe how these data from various sources yielded values for the relative risks and hazard ratios.

### Computation of variance for the hazard ratio for people under treatment

The hazard ratio for people under treatment was taken from Murphy and colleagues [23]. Because relative risks are found using a linear regression on a logarithmic scale, the variance of the parameter on a linear scale has to be estimated. The variance of the logarithm of a variable can be estimated using a second order Taylor series expansion. Thus, we can describe a hazard ratio for which only the variance of its natural logarithm is known as follows:

$$\begin{array}{l} Var[\ln(HR)]\;≅\;\frac{1}{HR^{2}}Var[HR]\\ \Rightarrow \;Var[HR]\;≅\;HR^{2}Var[\ln(HR)] \end{array}$$

### Computation of variance for the relative risk of death for nonadherence

The relative risk of death for people not adhering to treatment is only known as its logarithmic counterpart. Since the value taken from Lima and colleagues [11] represented the relative risk of people adhering to their treatment, the relative risk used in our formula requires the inverse of this parameter. The variance of the relative risk for nonadherence, RR_na_, can be derived from the variance of the logarithm of the relative risk of mortality for nonadherence using the following method:

$$\begin{array}{l} Var[\ln(RR_{a})]\;=\;Var\left[\ln\left(\frac{1}{RR_{na}}\right)\right]\;≅\\ \;{\left(RR_{na}\frac{-1}{RR_{na}{}^{2}}\right)}^{2}\;Var[RR_{na}]\;=\\ {\left(-\frac{1}{RR_{na}}\right)}^{2}Var[RR_{na}]\\ \Rightarrow \;Var[RR_{na}]\;≅\;RR_{na}{}^{2}Var\left[\frac{1}{\ln(RR_{na})}\right]\;=\\ RR_{na}{}^{2}Var[\ln(RR_{a})] \end{array}$$

where RR_a _is the relative risk of mortality for people who adhere to antiretroviral therapy.

### Computation of variance for relative risk of nonadherence due to alcohol

The odds ratio for adherence related to alcohol use provided by Hendershot and colleagues [18] can be transformed into a relative risk function following two simple steps:

• Take the inverse of the odds ratio to obtain the odds ratio of nonadherence for drinkers compared to nondrinkers.

• Compute the relative risk for nonadherence for drinkers versus nondrinkers.

The propagation of variance needs to be estimated for each of these steps. The variance of the first step can be approximated in the same manner as the hazard ratio. The relative risk for nonadherence of drinkers compared to nondrinkers is then calculated as follows:

$$RR\;=\;\frac{OR}{(1\;-\;P)\;+\;P\;\cdot \;OR}$$

where OR is the odds ratio of nonadherence for drinkers compared to nondrinkers, and P is the proportion of nonadherence among drinkers. Lacking the necessary information about this proportion, we took the proportion of nonadherence of the entire population as a conservative estimate of this value.

The variance of this relative risk can be computed using the Taylor series approximation with two variables as derived for the variance of PDT. In this case, the variance is given by:

$$Var[RR[OR,P]]\;≅\;D_{P}{}^{2}Var[P]\;+\;D_{OR}{}^{2}Var[OR]$$

with

$$D_{OR}\;=\;\frac{1\;-\;P}{{\left((1\;-\;P)\;+\;P\;\cdot \;OR\right)}^{2}}$$

and

$$D_{P}\;=\;\frac{OR(1\;-\;OR)}{{\left((1\;-\;P)\;+\;P\;\cdot \;OR\right)}^{2}}$$

The above relations define the relative risks, hazard ratio, and their respective variances, and allow us to compute the AAF of HIV/AIDS deaths.

## Results

**Figure 2:**
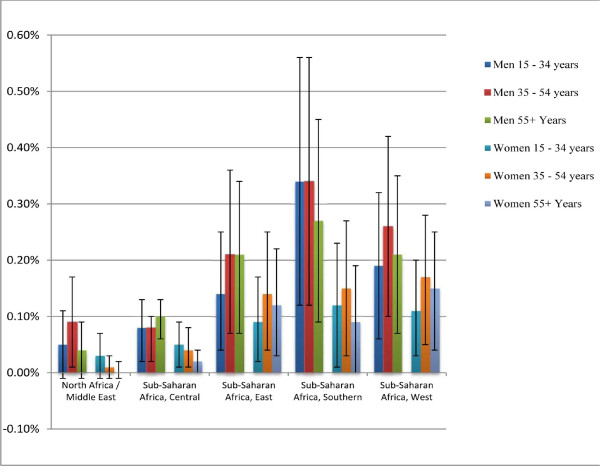
**Proportions of deaths caused by nonadherence to antiretroviral therapy due to alcohol consumption with their 95% uncertainty intervals for the five African GBD regions, by sex and age**.

Figure 2 shows the AAFs and their 95% uncertainty intervals for the five African GBD regions, separated by sex and age. Although the effect is small, an inspection of the uncertainty intervals shows that alcohol has a negative effect on the outcome of an HIV infection. Of the five African GBD regions, only the North Africa/Middle East region had strata where the 95% uncertainty intervals crossed 0, with only the men in that region aged 35 to 54 years exhibiting a significant positive AAF.

Overall, in Africa, men have higher AAFs than women, with AAFs generally highest in people under 55 years of age; however, not all of these differences are statistically significant. The highest AAFs are in sub-Saharan Africa, East, where the AAF was 0.34 (95% CI: 0.12, 0.56) for men aged 15 to 34 years, and 0.27 (95% CI: 0.09, 0.45) for men aged 35 to 54 years. In women, the highest AAFs are observed in the sub-Saharan Africa regions; however, unlike the men, the AAFs are very similar for the East, South, and West regions.

## Discussion

This method allows a systematic computation of partial AAFs for HIV/AIDS in any given population, including the 95% uncertainty intervals. Despite the limited information available, the results show a clear negative impact of alcohol on adherence to an antiretroviral treatment regime and consequently on the outcome of the disease, especially in the sub-Saharan regions of Africa. Although the AAFs are relatively low, HIV/AIDS was responsible for 58.5 million DALYs in 2004 [7], indicating that the burden of alcohol-attributable nonadherence to HIV/AIDS treatment is substantial.

The very broad categories used to describe alcohol consumption and adherence to antiretroviral therapy do not allow us to distinguish between moderate and heavy drinking or different percentages of nonadherence, and future research will be necessary to estimate a more accurate AAF for HIV/AIDS mortality. Ideally, AAFs would be estimated by using a continuous distribution of alcohol consumption and the risk relations for adherence to antiretroviral treatment associated with each level of consumption, as well as by specific patterns of drinking, such as heavy occasional drinking [30]. Modeling alcohol consumption with these two dimensions continuously and triangulating data obtained from surveys with adult per capita consumption information [31,32] are especially important for countries with high variation in patterns of drinking among drinkers.

These results may not be relevant for all parts of Africa. Many Africans completely abstain from alcohol, while many of those who drink tend to do so heavily, resulting in comparatively small variations in drinking patterns among drinkers http://apps.who.int/globalatlas/default.asp. Since we expect alcohol consumed as part of heavy drinking patterns to interfere more with antiretroviral treatment than moderate patterns of drinking [18,33], our estimates of AAFs based on the dichotomous variable of alcohol consumption (yes/no) constitute conservative estimates of the alcohol-attributable HIV/AIDS disease burden.

It is important to note that both the proportion of drinkers as well the proportion of people on antiretroviral therapy influence the AAF. Indeed, even though the proportion of drinkers in the sub-Saharan Africa, South region, is lower than in the sub-Saharan Africa, West region, the proportion of people in treatment is larger in the South region, which yields a larger overall AAF for this region. It is very likely that alcohol also has an effect on the progress of the disease in people without treatment that we did not estimate, but today's research does not allow us to deduce causality or quantify such an effect [6]. Improving this situation by conducting research to ascertain and quantify the effect of alcohol use on the disease progression of HIV/AIDS-related morbidity and mortality without antiretroviral treatment should be a priority.

## Conclusions

Although the presented method could be improved by better definition and quantification of exposure, it provides an evidence-based conservative estimate of the impact of alcohol use on HIV/AIDS mortality and highlights the importance of further research in this field. It also points to the need to integrate alcohol prevention measures [34] into the protocol for antiretroviral treatment.

## Competing interests

The authors declare that they have no competing interests.

## Authors' contributions

GG, KS, and JR conceptualized the overall article, contributed to the methodology, identified sources for risk relations and exposure, and contributed to the writing. GG was responsible for the derivations. All authors have approved the final version.

## Supplementary Material

Additional file 1 **Appendix 1 outlines the derivation of the AAF variance for HIV**. The derivations of the variance of the multiplication of two independent random variables and the derivations of the variances of AdAF, NAAAF, and PDT, using Taylor Series expansions for one or multiple variables, are described. Click here for file
