# Supplementary material for: Developing a method to derive alcohol-attributable fractions for HIV/AIDS mortality based on alcohol's impact on adherence to antiretroviral medication
Source: Popul Health Metr. 2011 Feb 14;9:5. doi: 10.1186/1478-7954-9-5 (PMC3048542; doi:10.1186/1478-7954-9-5)
Supplement: Additional file 1 — Appendix 1 outlines the derivation of the AAF variance for HIV. The derivations of the variance of the multiplication of two independent random variables and the derivations of the variances of AdAF, NAAAF, and PDT, using Taylor Series expansions for one or multiple variables, are described. [file 1478-7954-9-5-S1.PDF]

Appendix I

Variance of the multiplication of two distributions

For two independent variables X and Y, the variance can be derived as follows:

Equation (1)

$$\boxed{Var[XY]} = E[(XY)^2] - E[XY]^2 = E[X^2]E[Y^2] - E[X]^2E[Y]^2 =$$
$$= E[X^2]E[Y^2] - E[X]^2E[Y^2] + E[X]^2E[Y^2] - E[X]^2E[Y]^2 =$$
$$= Var[X]E[Y^2] + E[X]^2Var[Y] =$$
$$= \boxed{Var[X]Var[Y] + E[Y]^2Var[X] + E[X]^2Var[Y]}$$

Variance of AdAF and NAAAF

AdAF and the NAAAF (expressed below as an Attributable Fraction (AF)) are the same function with different parameters. An AF can be expressed as:

Equation (2):

$$AF = \frac{XY - X}{1 + XY - X}$$

Where X and Y are two independent variables. This can be simplified to:

Equation (3):

$$AF(Z) = \frac{Z}{1 + Z}$$

With Z=X(Y-1). When expressed as equation 3 the function depends on a single variable and can be approximated easily by a Taylor series expansion in one dimension under the assumption that the

function is sufficiently smooth. In general, around a point x_o any function of class C^{∞} can be written as:

Equation (4):

$$f(x) = \sum_{n=0}^{\infty} \frac{f^{(n)}(x_o)}{n!}(x - x_o)^n$$

Where $f^{(n)}(x_o)$ denotes the nth derivative of *f(x)* evaluated in x_o. This series is the Taylor series expansion of function *f* around point x_0.

Thus, the variance of a function *f(x)* of a variable X around its mean value μ_X can be derived by evaluating the definition of variance using the Taylor series expansion of function f. We will restrain our analysis to a second order expansion assuming all higher order terms to be negligible:

Equation (5):

$$Var[f(X)] \cong Var\left[f(\mu_X) + f'(\mu_X)(X - \mu_X) + \frac{1}{2}f''(\mu_X)(X - \mu_X)^2\right] =$$

$$= \frac{1}{N}\sum_{i=1}^{N}\left(f(\mu_X) + f'(\mu_X)(X_i - \mu_X) + \frac{1}{2}f''(\mu_X)(X_i - \mu_X)^2 - f(\mu_X)\right)^2 =$$

$$= \frac{1}{N}\sum_{i=1}^{N}\left(f'(\mu_X)(X_i - \mu_X) + \frac{1}{2}f''(\mu_X)(X_i - \mu_X)^2\right)^2 =$$

$$= \frac{1}{N}\sum_{i=1}^{N}\left(f'(\mu_X)^2(X_i - \mu_X)^2 + f'(\mu_X)f''(\mu_X)(X_i - \mu_X)^3 + \frac{1}{4}f''(\mu_X)^2(X_i - \mu_X)^4\right)$$

As we only take the second order Taylor series expansion, the 2 last terms of the above expression are dropped and we have:

Equation (6):

$$Var[f(X)] \cong \frac{1}{N}\sum_{i=1}^{N} f'(\mu_X)^2(X_i - \mu_X)^2 = f'(\mu_X)^2 Var[X]$$

This general expression can now be applied to our AF function as are twice differentiable thus insuring the first derivative to be continuous and we have:

Equation (7):

$$Var[AF(X)] \cong \frac{1}{(1+E[X])^4} Var[X]$$

By using equation (1) we can find the variances of AdAF and NAAAF as they are expressions of the same AF function.

For AdAF $= P_{na}(RR_{na}-1)$:

Equation (8):

$$\boxed{Var[AdAF(P_{na}, RR_{na})] \cong \frac{1}{\left(1+P_{na}(RR_{na}-1)\right)^4} Var[P_{na}RR_{na}]}$$

Where $Var[P_{na}RR_{na}] = Var[P_{na}]Var[RR_{na}] + {RR_{na}}^2 Var[P_{na}] + {P_{na}}^2 Var[RR_{na}]$

And for NAAAF with $X = P_{drink}(RR_{drink}-1)$:

Equation (9):

$$\boxed{Var[NAAAF(P_{drink}, RR_{drink})] = \frac{1}{\left(1+P_{drink}(RR_{drink}-1)\right)^4} Var[P_{drink}RR_{drink}]}$$

Where

$Var[P_{drink}RR_{drink}] = Var[P_{drink}]Var[RR_{drink}] + {RR_{drink}}^2 Var[P_{drink}] + {P_{drink}}^2 Var[RR_{drink}]$

Variance of PDT

The variance of the estimated proportion of HIV deaths for people currently under treatment cannot be expressed as a function of a single variable and therefore the Taylor series expansion has to be made in two-dimensions. The second order Taylor approximation for a function of two variables around a certain point in two-dimensional space $\boldsymbol{x_o}$ can be expressed as follows:

Equation (10):

$$f(\boldsymbol{x}) \cong f(\boldsymbol{x_o}) + (\boldsymbol{x} - \boldsymbol{x_o})^T \boldsymbol{\nabla} f(\boldsymbol{x_0}) + \frac{1}{2}[(\boldsymbol{x} - \boldsymbol{x_o})^T Hess(f(\boldsymbol{x_o}))(\boldsymbol{x} - \boldsymbol{x_o})]$$

Where letters in bold represent 2-dimensional vectors. $\boldsymbol{\nabla} f(\boldsymbol{x_0})$ represents the gradient and $Hess(f(\boldsymbol{x_o}))$ the Hessian matrix of function *f(x)* evaluated at point $\boldsymbol{x_o}$.

Using the above approximation the variance of function *f* around its mean value $\boldsymbol{\mu_x}$ can be approximated by:

Equation (11):

$$Var[f(\boldsymbol{X})] \cong Var\left[f(\boldsymbol{\mu_X}) + (\boldsymbol{X} - \boldsymbol{\mu_X})^T \boldsymbol{\nabla} f(\boldsymbol{\mu_X}) + \frac{1}{2}[(\boldsymbol{X} - \boldsymbol{\mu_X})^T Hess(f(\boldsymbol{\mu_X}))(\boldsymbol{X} - \boldsymbol{\mu_X})]\right] =$$

$$= \frac{1}{N}\sum_{i=1}^{N}\Big[f(\boldsymbol{\mu_X}) + (\boldsymbol{X}_i - \boldsymbol{\mu_X})^T \boldsymbol{\nabla} f(\boldsymbol{\mu_X}) + \frac{1}{2}[(\boldsymbol{X}_i - \boldsymbol{\mu_X})^T Hess(f(\boldsymbol{\mu_X}))(\boldsymbol{X}_i - \boldsymbol{\mu_X})]$$

$$- f(\boldsymbol{\mu_X})\Big]^2 = \frac{1}{N}\sum_{i=1}^{N}\left[(\boldsymbol{X}_i - \boldsymbol{\mu_X})^T \boldsymbol{\nabla} f(\boldsymbol{\mu_X}) + \frac{1}{2}[(\boldsymbol{X}_i - \boldsymbol{\mu_X})^T Hess(f(\boldsymbol{\mu_X}))(\boldsymbol{X}_i - \boldsymbol{\mu_X})]\right]^2$$

Considering only the terms of second order or less we have:

Equation (12)

$$Var[f(\boldsymbol{X})] \cong \frac{1}{N}\sum_{i=1}^{N}(\boldsymbol{X}_i - \boldsymbol{\mu_X})^T \nabla f(\boldsymbol{\mu_X})(\boldsymbol{X}_i - \boldsymbol{\mu_X})^T \nabla f(\boldsymbol{\mu_X})$$

$$= \frac{1}{N}\sum_{i=1}^{N}\left[\left[\frac{\partial}{\partial X_1} f(\boldsymbol{X} = \boldsymbol{\mu_X})\right]^2 (X_1 - \mu_{X_1})^2 + \left[\frac{\partial}{\partial X_2} f(\boldsymbol{X} = \boldsymbol{\mu_X})\right]^2 (X_2 - \mu_{X_2})^2\right.$$

$$\left. + 2\frac{\partial}{\partial X_1} f(\boldsymbol{X} = \boldsymbol{\mu_X}) \frac{\partial}{\partial X_2} f(\boldsymbol{X} = \boldsymbol{\mu_X})(X_1 - \mu_{X_1})(X_2 - \mu_{X_2})\right]$$

$$= \left[\frac{\partial}{\partial X_1} f(\boldsymbol{X} = \boldsymbol{\mu_X})\right]^2 Var[X_1] + \left[\frac{\partial}{\partial X_2} f(\boldsymbol{X} = \boldsymbol{\mu_X})\right]^2 Var[X_2]$$

$$+ 2\,\frac{\partial}{\partial X_1} f(\boldsymbol{X} = \boldsymbol{\mu_X})\,\frac{\partial}{\partial X_2} f(\boldsymbol{X} = \boldsymbol{\mu_X}) Covar[X_1, X_2]$$

With X_1 and X_2 the two variables making up the vector **X**, and $\frac{\partial}{\partial X_i} f(\boldsymbol{X} = \boldsymbol{\mu_X})$ the partial derivative with respect to the i-th component of vector **X** evaluated at point $\boldsymbol{\mu_X}$. In our calculation of PDT, the variables X_1 and X_2 are respectively P_{treat} and HR_{treat} and therefore independent. Thus, the above expression simplifies to:

Equation (13):

$$\boxed{Var\left[PDT[P_{treat}, HR_{non-treat}]\right] \cong D_{P_{treat}}{}^2 Var[P_{treat}] + D_{HR_{non-treat}}{}^2 Var[HR_{non-treat}]}$$

Where D_{Ptreat} and $D_{HRnon\text{-}treat}$ are the partial derivatives of the PDT function with respect to P_{treat} and $HR_{non\text{-}treat}$. These partial derivatives are given by:

$$D_{P_{treat}} = \frac{HR_{non-treat}}{(P_{treat} + HR_{non-treat}(1 - P_{treat}))^2}$$

$$D_{HR_{non-treat}} = \frac{P_{treat}(1 - P_{treat})}{(P_{treat} + HR_{non-treat}(1 - P_{treat}))^2}$$
